# Achievement of a Parameter Window for the Selective Laser Melting Formation of a GH3625 Alloy

**DOI:** 10.3390/ma17102333

**Published:** 2024-05-14

**Authors:** Guozheng Quan, Qi Deng, Yifan Zhao, Mingguo Quan, Daijian Wu

**Affiliations:** 1 Chongqing Key Laboratory of Advanced Mold Intelligent Manufacturing, School of Material Science and Engineering, Chongqing University, Chongqing 400044, China; dengqi77929@sina.com (Q.D.); z1147964348@163.com (Y.Z.); quanmg2258@sina.com (M.Q.); wdjxhu@163.com (D.W.); 2 Jiangsu Yutaida Industrial Technology Co., Ltd., Taizhou 225300, China; 3 Huan Ding Intelligent Technology (Suzhou) Co., Ltd., Suzhou 215000, China; 4 Sichuan Laboratory of Advanced Manufacturing Technology of Press Engine, Sichuan Polytechnic University, Deyang 618000, China

**Keywords:** selective laser melting, molten pool morphology, GH3625 alloy, parameter window

## Abstract

In the selective laser melting (SLM) process, adjusting process parameters contributes to achieving the desired molten pool morphology, thereby enhancing the mechanical properties and dimensional accuracy of manufactured components. The parameter window characterizing the relationship between molten pool morphology and process parameters serves as an effective tool to improve SLM’s forming quality. This work established a mesoscale model of the SLM process for a GH3625 alloy based on the discrete element method (DEM) and computational fluid dynamics (CFD) to simulate the forming process of a single molten track. Subsequently, the formation mechanism and evolution process of the molten pool were revealed. The effects of laser power and scanning speed on the molten pool size and molten track morphology were analyzed. Finally, a parameter window was established from the simulation results. The results indicated that reducing the scanning speed and increasing the laser power would lead to an increase in molten pool depth and width, resulting in the formation of an uneven width in the molten track. Moreover, accelerating the scanning speed and decreasing the laser power cause a reduction in molten pool depth and width, causing narrow and discontinuous molten tracks. The accuracy of the simulation was validated by comparing experimental and simulated molten pool sizes.

## 1. Introduction

The GH3625 nickel-based superalloy is widely used in the thermal sections of aircraft engines and in gas turbines for power plants due to its excellent mechanical properties, oxidation, and corrosion resistance [1,2,3]. With the pursuit of lightweight and high fatigue strength parts, the structure of components is evolving towards integration. The external shape and internal structure of such components are becoming increasingly complex, making traditional methods such as casting and forging more challenging to use for their manufacture [4]. Selective laser melting (SLM), considered an additive manufacturing technology, possesses relatively high material utilization and the capability to manufacture complex structures without the need for molds, unlike traditional forming methods [5,6]. The basic principle of the SLM process is to shape a single layer through the overlay of single molten tracks and then form the entire component layer by layer. The accuracy of the accumulated dimensions in this process is highly dependent on the precise control of the molten pool’s morphology [6,7]. An excessive depth to the molten pool can result in a keyhole shape, which can hinder vapor escape and increase the porosity of the component [8,9,10]. Meanwhile, shallow molten pools can result in insufficient melting, which also leads to high porosity and poor mechanical properties [11,12]. It is imperative to ensure the uniform, smooth, and stable morphology of the molten pool.

The optimization of processing parameters contributes to achieving the desired molten pool morphology in the SLM process. A deep understanding of the relationship between process parameters and molten pool morphology is a prerequisite for conducting this optimization. Liu et al. [13] established a mathematical model based on Smoothed Particle Hydrodynamics (SPH) to examine the influence of surface tension on molten pool morphology. Their study investigated the formation mechanism of the longitudinal morphology of the molten pool and explored the variations in molten pool length and depth under different process parameters. Yang L X et al. [14] investigated the corresponding relationship between molten pool morphology, scanning speed, and surface tension by establishing equilibrium equations for surface tension, gravity, and external forces. Studies conducted by Adjamsky et al. [15] on Inconel 718 nickel alloy and Raut et al. [16] on IN625 investigated the relationship between process parameters and molten pool morphology and macro-mechanical properties. The above research demonstrates that the relationship between process parameters and molten pool morphology can be elucidated through experimentation and numerical simulations.

Due to the extremely small size of the molten pool in the SLM process and the extremely short melting and solidification times of the powder, the experimental exploration of the molten pool’s evolution and the influence of process parameters on molten pool morphology becomes an expensive and time-consuming process. Numerical simulation is an efficient and cost-effective analysis technique that has been applied to model the SLM process [17]. Francois et al. [18] posit that the interaction between the laser and metal materials occurs at the mesoscale. Mesoscale models considering complex thermophysical phenomena such as melt flow, solidification, and heat transfer provide a realistic simulation of the evolution of the molten pool and the influence of process parameters on molten pool morphology. The current utilization of mesoscale models for analyzing the SLM process is limited. This limitation arises from the challenges faced by many simulation softwares, which struggle to directly simulate the complex thermophysical phenomena associated with the SLM process, often requiring additional development or source code modifications. Moreover, the small grid size of mesoscale models contributes to their high calculation times [19]. Some researchers have employed simplified mesoscale models to investigate molten pool behaviors. For example, Verhaeghe et al. [20] studied the effects of metal evaporation on molten pool morphology using a simplified mesoscale model. Their model primarily considered the evaporative effect while neglecting the influence of other molten pool driving forces. Based on this, Heeling et al. [21] incorporated surface tension and thermal capillary forces into a simplified mesoscale model to investigate molten pool flow during the SLM of an IN738LC alloy. Their research indicated that the recoil forces from evaporation play a crucial role in molten pool behaviors. Similarly, Dai et al. [22] and Zhang et al. [23] found that the recoil forces induced by evaporation significantly affect molten pool morphology, causing a depression at the leading edge of the molten pool. However, simplified mesoscale models neglect the influence of powder particles, and the accuracy of their research results needs improvement. With the development of mesoscale models, researchers have started to utilize them to study molten pool behaviors. Zheng et al. [24] created a coupled mesoscale model using the height function–lattice Boltzmann method (HF-LBM). In this model, a novel interface capture technique was employed to simulate melt flow by considering interface forces such as surface tension, Marangoni convection, and recoil pressure. Studies conducted by Liu et al. [25], Lu et al. [26], and Azadi et al. [27] established mesoscale models to investigate the evolution of molten pools. These models utilized a randomly filled powder bed generated based on the discrete element method (DEM) and employed a fluid volume method based on computational fluid dynamics (CFD) to examine the molten pool’s surface. In summary, it is feasible to use mesoscale models to simulate molten pool evolution and establish the relationship between process parameters and molten pool morphology.

**Figure 1:**
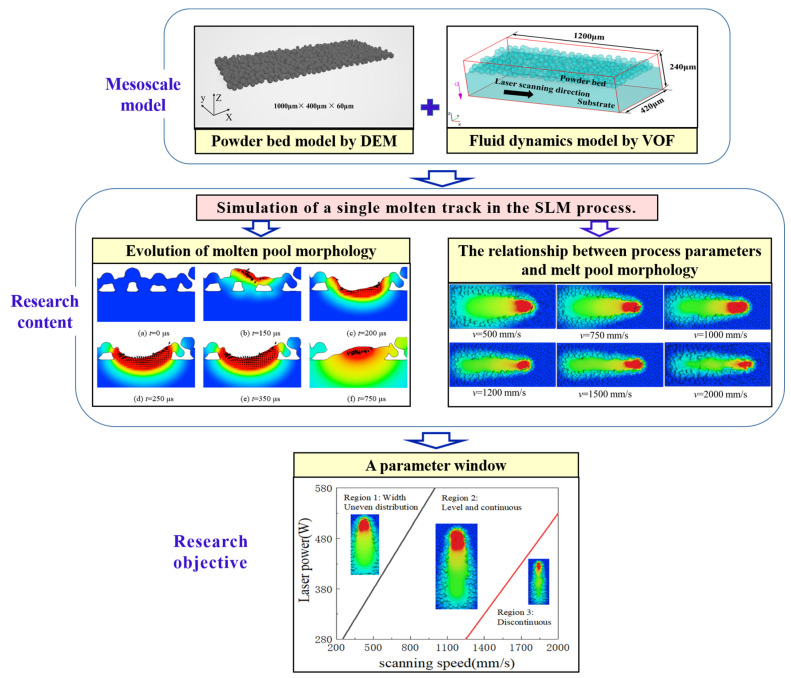
The method for obtaining the parameter window.

In this work, a parameter window of the GH3625 alloy during the SLM process, depicting the relationships between the molten pool morphology and process parameters, was established using mesoscale numerical simulations. The method for constructing this window is exhibited in Figure 1. Firstly, a mesoscale model was established based on the discrete element method (DEM) and computational fluid dynamics (CFD). Subsequently, the evolution of the molten pool’s morphology during the single molten track formation process was simulated. The effects of laser power and scanning speed on the molten pool size and molten track morphology were analyzed. Finally, the parameter window for the GH3625 alloy was established from the perspective of the molten pool’s morphology, and the optimal SLM range of the process parameters for the GH3625 alloy was identified. The accuracy of the simulation was verified by comparing the simulated and experimental values of the molten pool’s size. This study provides guidance for the SLM production of GH3625 alloy components.

## 2. Numerical Simulation

During the SLM process, the laser continuously radiates the powder particles to form a mesoscopic transient molten pool, which undergoes complex phase transition processes such as melting, evaporation, and solidification. Mesoscale models considering complex thermophysical phenomena such as melt flow, solidification, and heat transfer provide a realistic simulation of the evolution of the molten pool and the influence of process parameters on the molten pool’s morphology. The model includes a randomly filled powder bed model based on the discrete element method (DEM) and a fluid dynamics model based on computational fluid dynamics (CFD).

### 2.1. Powder Bed Modeling in Mesoscopic Model

**Figure 2:**
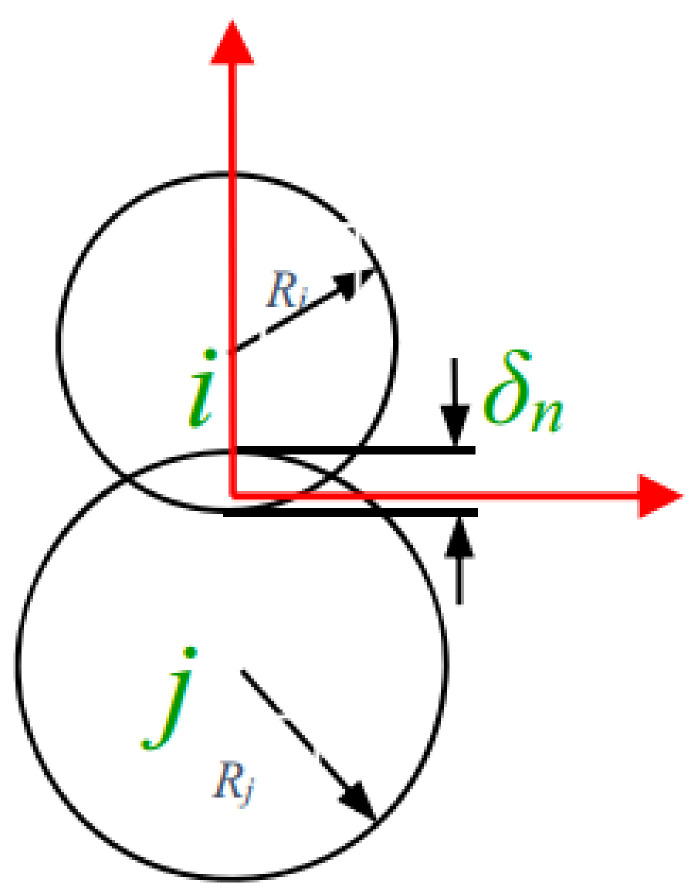
Model of intergranular force [30].

DEM is a simulation method used to study the macroscopic motion of complex, non-continuous media by their analyzing particle forces and displacements. During the generation of the powder bed, the motion state of its particles was governed by Newton’s second law, and the contact model between particles was set as the Hertz–Mindlin (no-slip) model. This model calculates the contact force based on the normal overlap and tangential displacement between particles. The measurement of inter-particle force is shown in Figure 2. The normal force component follows the Hertz contact theory [28], and the tangential force is modeled using the Mindlin–Deresiewicz theory [29]. All contact forces can be determined as follows:

(1) $$F_{n}=\frac{4}{3}E^{*}\sqrt{R^{*}}\delta_{n}^{1.5}$$

(2) $$\frac{1}{E^{*}}=\frac{\left(1-v_{i}^{2}\right)}{E_{i}}+\frac{\left(1-v_{j}^{2}\right)}{E_{j}}$$

(3) $$\frac{1}{R^{*}}=\frac{1}{R_{i}}+\frac{1}{R_{j}}$$

(4) $$F_{t}=-S_{t}\delta_{t}=-8G^{*}\sqrt{R^{*}\delta_{n}}$$

where *F_n_* and *F_t_* are the forces in the normal and tangential direction, respectively. E*, R*, G*, *S_t_*, *δ_n_*, and *δ_t_* are the equivalent Young’s modulus, equivalent radius, equivalent shear modulus, tangential stiffness, normal overlap, and tangential overlap. *E_i_*, *v_i_*, and *R*_i_ and *E_j_*, *v_j_*, and *R_j_* are defined as the Young’s modulus, Poisson’s ratio, and radius of the contact spheres *i* and *j*, respectively.

**Figure 3:**
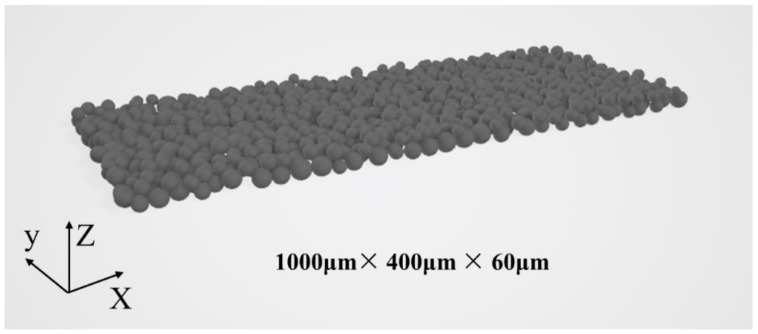
Powder bed filler model.

The powder material is spherical and has a particle size of 15–53 μm, with a normal distribution of diameters. Generating a powder bed through DEM involves several steps: initially, a cloud of metal powder is formed above the substrate. Subsequently, powder particles descend onto the substrate due to gravity, accumulating freely to create the powder bed. The layer thickness is then set to 60 μm, and any particles on the powder bed surface exceeding this height are removed, resulting in the initial powder bed model. This method produces a powder bed closely resembling experimental conditions. The main mechanical properties used for the DEM simulation included a Young’s modulus of 207 GPa and a friction coefficient between particles of 0.3. The powder bed model is illustrated in Figure 3. Subsequently, the powder bed model was converted into a stl file and imported into the CFD model for further configuration.

### 2.2. Fluid Dynamics Modeling in the Mesoscopic Model

#### 2.2.1. Heat Transfer and Fluid Flow Equations

A CFD model simulating the complex thermophysical phenomena occurring during the SLM process has been established. To streamline computations, it is assumed in the model that the fluid in the molten pool is incompressible and Newtonian, with a laminar flow. The equations for mass, momentum, and energy conservation in the model are solved as follows [31].

Mass:

(5) $$\nabla \cdot \vec{v}=0$$

Momentum:

(6) $$\frac{\partial \vec{v}}{\partial t}+\left(\vec{v}\cdot \nabla \right)\vec{v}=-\frac{1}{\rho }\nabla p+\mu \nabla^{2}\vec{v}+\vec{g}\left[1-\beta \left(T-T_{m}\right)\right]$$

Energy:

(7) $$\frac{\partial h}{\partial t}+\left(\vec{v}\cdot \nabla \right)h=\frac{1}{\rho }\left(\nabla \cdot \lambda \nabla T\right)$$

where *v* is the fluid velocity of the molten pool, *t* is the time, *p* is the pressure, *µ* is the viscosity, *g* is the gravitational acceleration, *β* is the coefficient of thermal expansion, *T* is the temperature, *T_s_* is the melting temperature of the material, *h* is the enthalpy, *ρ* is density, and *λ* is the thermal conductivity of the material.

The forming process of a single molten track was numerically simulated using a CFD model in this paper. The volume-of-fluid (VOF) method was utilized to track variations in the surface shape of the molten track and capture its morphology. The expression of the momentum equation for the change at the interface is as follows [32]:

(8) $$\frac{\partial F}{\partial t}+\vec{v}\cdot \nabla F=0$$

where *F* is the volume fraction of the nth phase, *t* is the time, and *v* is the fluid velocity.

*F* = 0 means that the mesh is empty and there is no fluid; *F* = 1 means that the mesh is completely occupied by a liquid phase at this time; and 0 < *F* < 1 means an interface between a fluid and a void.

To simulate the melting and solidification process in SLM, an enthalpy–porosity method is incorporated into the model to achieve a phase transition from solid to liquid phase, accounting for the latent heat of melting. The enthalpy–porosity method is as follows [33]:

(9) $$h=\int c_{p}dT+L_{f}f$$

where *c_p_* is the specific heat, *L_f_* is the latent heat of melting, and *f* is the volume fraction of liquid, determined by the temperature [34].

(10) $$\begin{array}{ll} f=\left\{\begin{array}{l} 0\\ \frac{T-T_{m}}{T_{L}-T_{m}}\\ 1 \end{array}\right. & \begin{array}{l} T-T_{m}\\ T_{m}\leq T\leq T_{L}\\ T-T_{L} \end{array} \end{array}$$

where *T_m_* is the solidus temperature, and *T_L_* is the liquidus temperature.

The fluid volume fraction’s field was obtained by solving the VOF equation concurrently with conservation Equations (5)–(7). To ensure an accurate representation of fluid-free surfaces in simulations, it is imperative to account for the effects of free boundary conditions, including surface tension and surface pressure conditions. The simulation of the Marangoni effect induced by the surface tension gradient is enabled by balancing shear stress as a free boundary condition, as described by Hyun Min et al. [35].

(11) $$-\mu \frac{\partial v_{l}}{\partial n}=\frac{\partial \gamma }{\partial t}\frac{\partial T}{\partial r}$$

where *μ* is the dynamic viscosity, ∂*γ*/∂*t* is the surface tension gradient, *v_l_* is the tangential velocity vector, *n* is the normal to the free surface, and *r* is the tangential direction on the free surface. The formulation of surface pressure as a boundary condition on the free surface can be expressed as [35]

(12) $$-p+2\mu \frac{\partial v_{n}}{\partial n}=-p_{r}+\gamma \left(\frac{1}{R_{x}}+\frac{1}{R_{y}}\right)$$

where *p* is the pressure, *v_n_* is the normal velocity vector, *P_r_* is the recoil pressure, *γ* is the surface tension, and *R_x_* and *R_y_* are the principal radii of the surface’s curvature. According to the work of Sahoo et al. [36], the surface tension of liquid metal exhibits a negative correlation with temperature, as described by the simplified formula

(13) $$\gamma (T)==\gamma_{m}+\frac{d\gamma }{dT}\left(T-T_{m}\right)$$

where γ(*T*) is the surface tension, *γ*_m_ is the surface tension when the material reaches its melting point, and *dγ*/*dT* is the temperature coefficient of surface tension. *T_m_* is the melting temperature. The recoil pressure model developed by Semak V [37] is widely embraced, as presented by

(14) $$P_{r}=0.54P_{0}\exp\left(\Delta H_{v}\frac{T-T_{v}}{RTT_{v}}\right)$$

where *P*_0_ is the ambient pressure, *T_v_* is the boiling temperature, Δ*H_v_* is the effective enthalpy of metal vapor, and *R* is the universal gas constant.

#### 2.2.2. Boundary Condition

The selection of the heat source function determines the form of the energy input, including the distribution and transfer of energy during the simulation of the temperature field in SLM formation. A Gaussian surface distribution heat source is employed in this study, providing an effective representation of the thermal flux density distribution on the material’s surface. This approach is especially suitable for the forming of thin sheet materials. The heat source is loaded on the surface of the powder bed, and the core of the heat source has the maximum heat flux density. The laser energy absorbed by the surrounding melt and powder is dependent on the spatial coordinates of the heat source’s position, as shown in the following formula [38,39]:

(15) $$q_{r}=\frac{2\eta P}{\pi R^{2}}\exp\left(-\frac{2r^{2}}{R^{2}}\right)$$

where the *q_r_* is the heat flux density at a point within the radius of the Gaussian laser beam, *P* is the total laser power, *η* is the laser absorption rate of the powder, *R* is the radius of the Gaussian laser beam, and *r* is the distance between a point within the radius of the Gaussian laser beam and the center of the heat source at any time. In general, the laser absorptivity of the powder bed is reported to be 3–5 times higher than that of the corresponding bulk material, as documented by Ang et al. [40]. and Stacy et al. [41]. The value of *η* in this study was taken to be 0.38 based on the relevant literature on nickel-based superalloys [31].

Due to the intricate energy exchange between the free interface and argon gas, the thermal boundary condition at the free interface is defined as follows [42,43,44]:

(16) $$q_{\mathrm{in}}=q-q_{\mathrm{con}}-q_{\mathrm{rad}}-q_{\mathrm{evap}}$$

where *q*_in_ is the change in heat flow across the molten pool’s free surface, and *q*_con_, *q*_rad_, and *q*_evap_ represent convection, radiation, and vaporization heat loss, respectively. The formulas for the three types of heat loss can be expressed as follows [45]:

(17) $$q_{\mathrm{con}}=h_{\mathrm{con}}\left(T-T_{\mathrm{ref}}\right)$$

(18) $$q_{\mathrm{rad}}=\sigma_{s}\varepsilon \left(T^{4}-T_{\mathrm{ref}}^{4}\right)$$

(19) $$q_{\mathrm{evap}}=0.82\frac{\Delta H_{v}}{\sqrt{2\pi MRT}}P_{0}\exp\left(\frac{\Delta H_{v}\left(T-T_{v}\right)}{RTT_{v}}\right)$$

where *h*_con_ is the convective heat transfer coefficient, *T*_ref_ is the ambient temperature, *σ_s_* is the Stefan–Boltzmann constant, *ε* is the emissivity, and *M* is the molar mass.

#### 2.2.3. CFD Model for SLM Process

**Figure 4:**
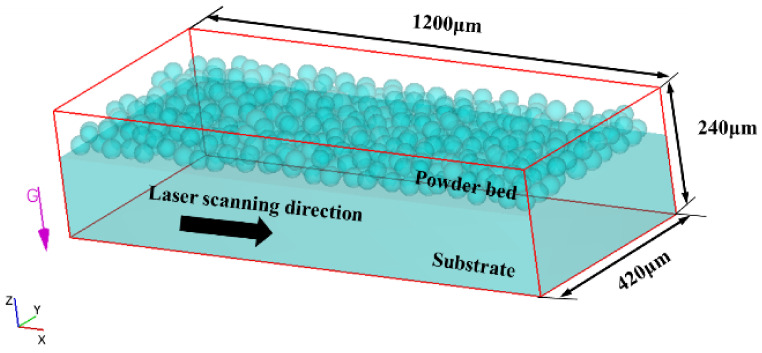
CFD model and calculation domains of SLM.

**Figure 5:**
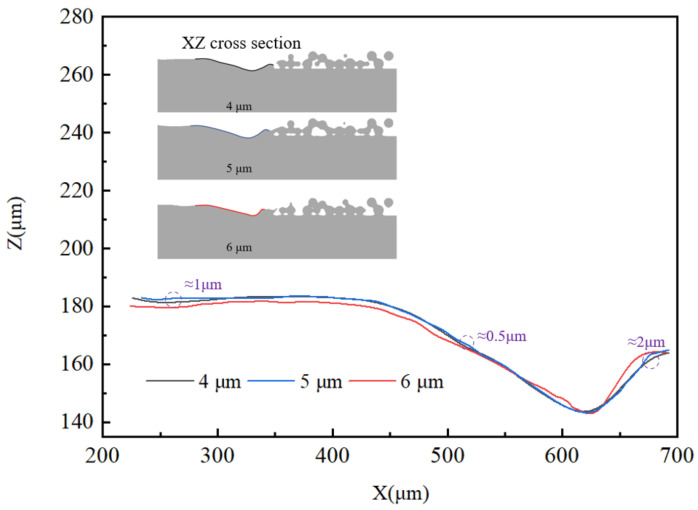
Cross-section contours of a molten track using three grid sizes.

The CFD model consists of a powder bed model (1000 µm× 400 µm× 60 µm) and a substrate (1200 µm× 420 µm× 150 µm), as shown in Figure 4. To ensure the accuracy of the simulation, the surface space of the powder bed needs to be meshed. The final overall size of the computational domain is 1200 µm× 420 µm× 240 µm. A convergence study on grid size was conducted to ensure computational efficiency and accuracy in simulations. Prior numerical investigations into fluid dynamics have suggested that maintaining a particle size eight times larger than the grid size is essential for ensuring the accuracy of simulation results [46]. Given the particle size range of 15–53 μm and the concern over computational load, three grid sizes (4 μm, 5 μm, and 6 μm) were chosen. Figure 5 shows the molten pool contours of the XZ cross-section using these three grid sizes under the same conditions. It is evident that the contours share a similar trend, with the contours of the 4 μm and 5 μm grid sizes overlapping well, exhibiting a maximum difference not exceeding 2 μm. However, the 6 μm grid size contours exhibit noticeable differences compared to those of the 4 μm and 5 μm grid sizes. As is well-known, numerical accuracy correlates with the number of grids, but excessive grids raise computational costs [47,48]. Therefore, ensuring numerical simulation precision while maintaining an appropriate number of grids is advantageous for cost savings. This paper proceeds with a 5 μm grid size to balance precision and efficiency.

### 2.3. Mesoscopic Model Setup

The model boundary conditions have two main aspects. On the one hand, the molten pool driving force theory is used as the boundary condition for the mesoscale model. On the other hand, we must set the physical boundary conditions for the entire computational domain. The upper surface is set as a pressure boundary condition, the surrounding surfaces are set as continuative boundary conditions, and the lower surface is set as a wall boundary condition for fixed constraints.

**Figure 6:**
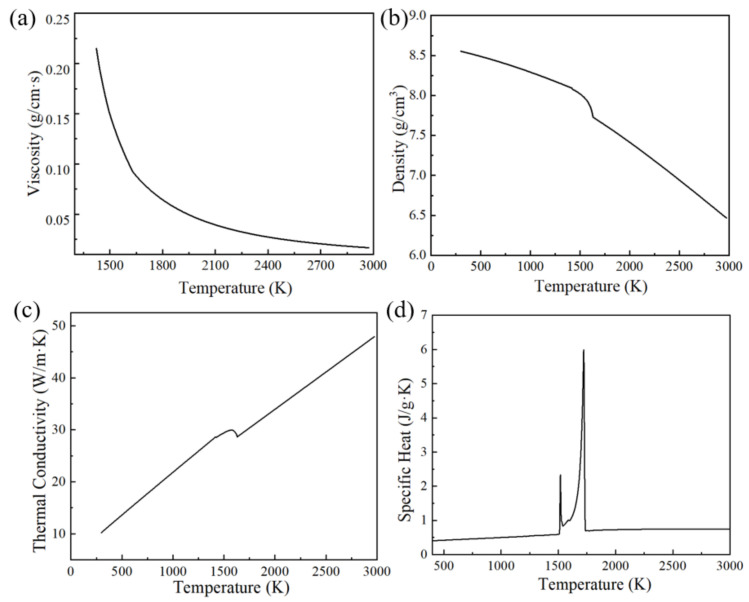
Thermophysical properties of GH3625: (**a**) viscosity, (**b**) density, (**c**) thermal conductivity, (**d**) specific heat.

This study employs the GH3625 alloy, and its chemical composition is shown in Table 1. Its viscosity, mass density, thermal conductivity, and specific heat were calculated using JmatPro software 11.2, as depicted in Figure 6. The processing parameters and material properties used in this paper are shown in Table 2. The printing device used in this study has a maximum power of 500 W. However, a simulated maximum power of 580 W was employed to explore the extreme conditions affecting molten pool morphology.

## 3. Evolution of Molten Pool Morphology during Single Molten Track Forming

### 3.1. Experimental Validation

Experiments on the SLM of the GH3625 alloy were carried out to verify the established mesoscale model. The experiments were conducted using LiM-X400 equipment from Tianjin Lei ming Laser Technology (Tianjin, China). A single-track trajectory of a 10 mm length was printed using a laser power of 380 W and a scanning speed of 1000 mm/s. To compare the sizes of the molten pools, the cross-section of the single tracks was polished and subsequently etched. The etchant consisted of 22 mL of HNO_3_ and 6 mL of HCl. Digital microscopy was employed to observe the track’s morphology from the top view and examine the metallographic images of its cross-sectional view.

**Figure 7:**
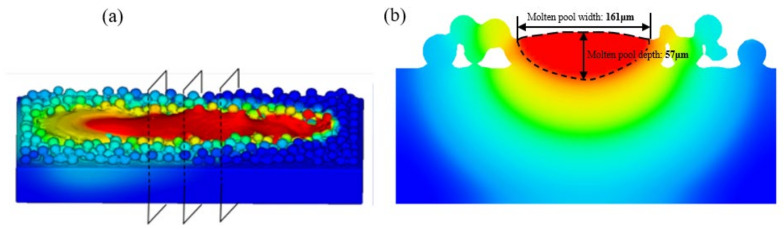
The simulated molten pool: (**a**) the three-dimensional schematic and (**b**) cross-section of ZY.

**Figure 8:**
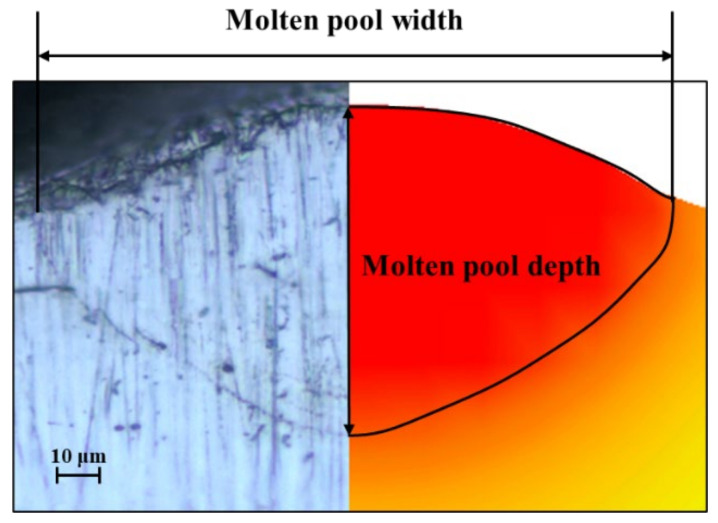
Experimental versus simulated molten pool size comparison.

To validate the accuracy of the simulation, the width and depth of the molten pool were measured on ZY sections at three positions along the molten track, as shown in Figure 7a. The red area in the ZY section represents the region above the melting temperature, where a liquid molten pool was formed. The simulated average width of the molten pool was 161 μm, and the depth was 57 μm, as depicted in Figure 7b. Figure 8 illustrates the comparison between experimental and simulated molten pool sizes. Experimental results indicate a molten pool width of 151 μm and a depth of 69 μm. The maximum error in molten pool width and depth between the experiment and simulation is less than 7%. Therefore, the proposed numerical model can predict the three-dimensional morphology of molten pools very accurately.

### 3.2. Forming Mechanism of the Molten Pool

**Figure 9:**
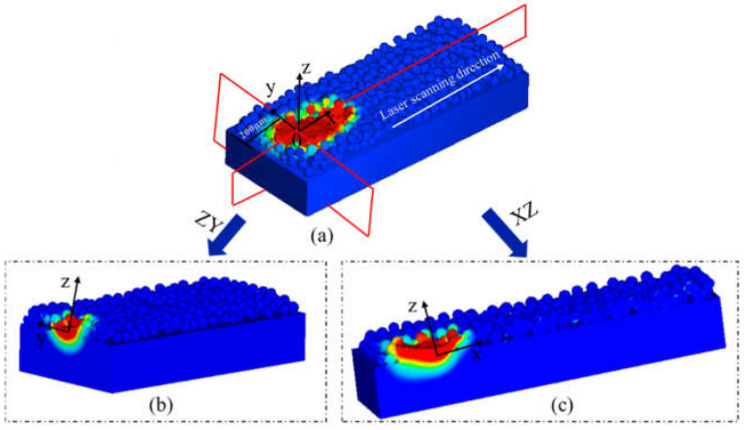
Schematic diagrams of (**a**) the three-dimensional model, (**b**) the cross-section of ZY, (**c**) the cross-section of XZ.

**Figure 10:**
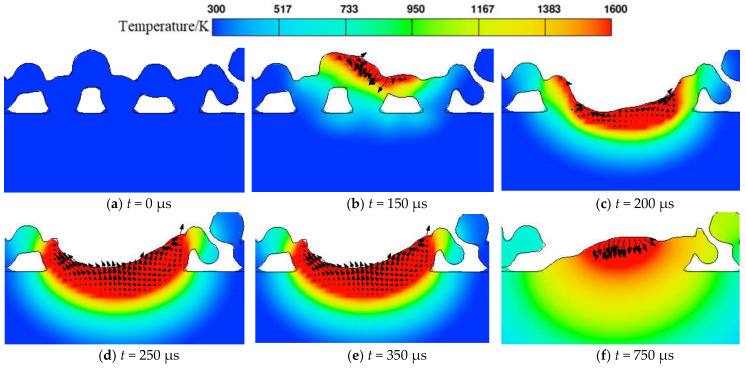
Evolution of molten pool during melting and solidification.

Figure 9 displays characteristic cross-sections of ZY and XZ, where the ZY cross-section is x = 200 μm from the laser starting point and the XZ cross-section is a section through the center of the Y-axis. Figure 10 illustrates the temperature field changes occurring during the melting and solidification process of powder particles in the ZY cross-section. The black arrow represents the velocity vector of the melt. The simulation results were obtained using a laser power of 380 W, a scanning speed of 1000 mm/s, and a powder layer thickness of 60 μm.

(a) The cross-section is the original shape of the particles, and there are more voids between the particles. (b) When *t* = 150 μs, the center of the laser heat source does not reach X = 200 μm, but it causes the particles to begin melting. (c) When *t* = 200 μs, the center of the laser heat source irradiates the cross-section, and the powder particles are completely melted, forming a concave molten pool. This concave morphology is formed due to the vapor recoil force induced by the instantaneous temperature exceeding boiling point at the center of the molten pool, driving the melt material downwards and increasing the depth of the molten pool [49,50]. (d)~(e) When *t* = 250 μs to 350 μs, the center of the laser heat source moves away from the cross-section, and the molten pool gradually flows to form a hill shape. At this stage, significant temperature gradients are induced by the convective and radiative heat exchange behaviors between the high-temperature molten pool and the surrounding environment. Significant temperature gradient changes result in variations in the surface tension gradients, leading to the Marangoni effect. The Marangoni effect causes the molten metal to flow into the surrounding low-temperature region. (f) When *t* = 750 μs, the molten pool has solidified into a hill shape, with reduced width and depth compared to Figure 10e. Furthermore, as the laser heat source’s center moves away from the cross-section, the weakening Marangoni effect, coupled with the combined influence of surface tension and gravity, results in a reduction in the height of the molten track.

**Figure 11:**
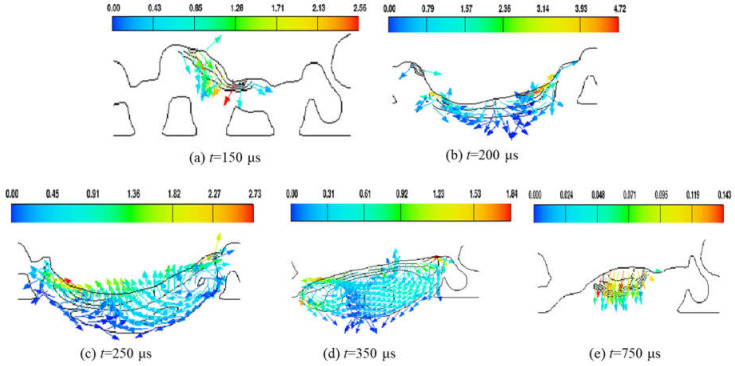
Evolution of liquid metal flow velocity field in ZY cross-section.

The molten pool flow field affects the morphology of the molten pool and its track after solidification. The molten pool flow is mainly influenced by the temperature field, with a material viscosity inversely proportional to its temperature. Figure 11 shows the evolution of the liquid-phase metal flow velocity field in the ZY cross-section. The direction of the arrows indicates the flow direction of the molten metal, while the color corresponds to the magnitude of the flow velocity. (a) When *t* = 150 μs, powder melting initiates and, under the influence of gravity and surface tension, molten metal flows downward, leading to the gradual fusion of the molten metal with the substrate to form a molten pool. (b) When *t* = 200 μs, the heat source center irradiates this cross-section, resulting in the highest temperature in the molten pool, leading to a decrease in the viscosity of the liquid metal and improved fluidity. Under the influence of vapor recoil forces, the flow direction of the molten metal is downward, resulting in an increase in the concave depth of the molten pool. (c) When *t* = 250 μs, it is observed that molten metal flows toward the center of the molten pool, as indicated by the arrows, resulting in a reduction in the depth of the depression. (d) When *t* = 350 μs, the metal flow in the molten pool is influenced by Marangoni convection and gravity, giving rise to a complex flow pattern. (e) When *t* = 750 μs, the molten pool has solidified and, due to the extremely poor fluidity of the material, minimal flow occurs solely under the influence of surface tension and gravity. The downward flow of the molten metal at this point results in a decrease in the heights of both the molten pool and track.

### 3.3. Evolution Process of Molten Pool

**Figure 12:**
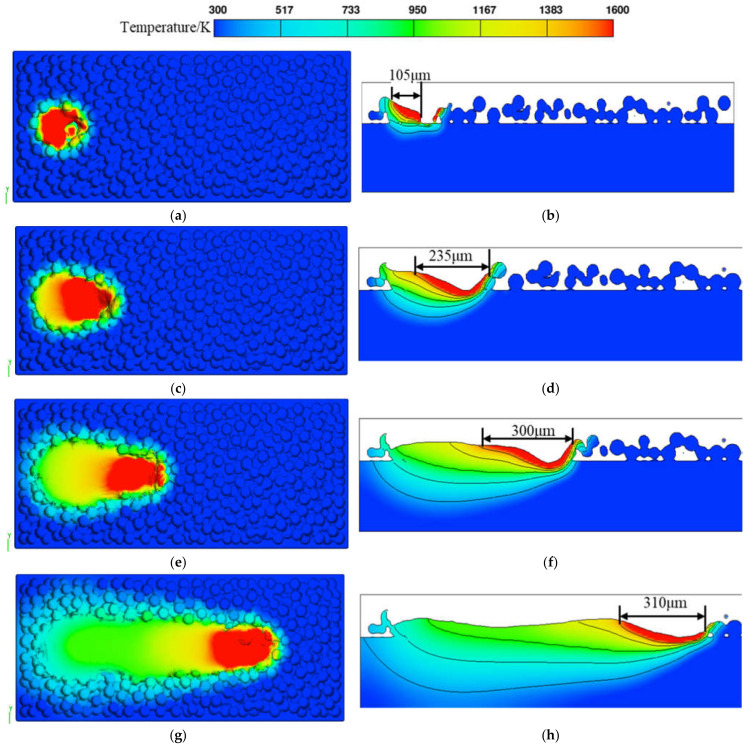
Temperature evolution of powder bed. (**a**) Three-dimensional view at *t* = 50 μs, (**b**) the XZ cross-section at *t* = 50 μs, (**c**) three-dimensional view at *t* = 150 μs, (**d**) the XZ cross-section at *t* = 150 μs, (**e**) three-dimensional view at *t* = 300 μs, (**f**) the XZ cross-section at *t* = 300 μs, (**g**) three-dimensional view at *t* = 650 μs, (**h**) the XZ cross-section at *t* = 650 μs.

When the temperature exceeds the melting point of GH3625, this material transitions into a liquid phase and forms a molten pool. Figure 12 illustrates the powder bed and XZ cross-sectional temperature field distribution of the single molten track trajectory of the GH3625 alloy. As the laser heat source moves forward, the molten pool and the heat-affected zone lengths progressively increase. The increase in the length of the heat-affected zone is due to the increased energy input causing heat conduction between the molten pool, unmelted metal, and the substrate, thereby preheating the powder in the unscanned region. The increase in molten pool length is attributed to the powder melting rate exceeding the solidification rate, causing the recently melted liquid metal to flow toward the already scanned region. Therefore, the shape of the molten pool gradually transitions from circular to elliptical, ultimately forming a comet shape with the increase in scanning time.

**Figure 13:**
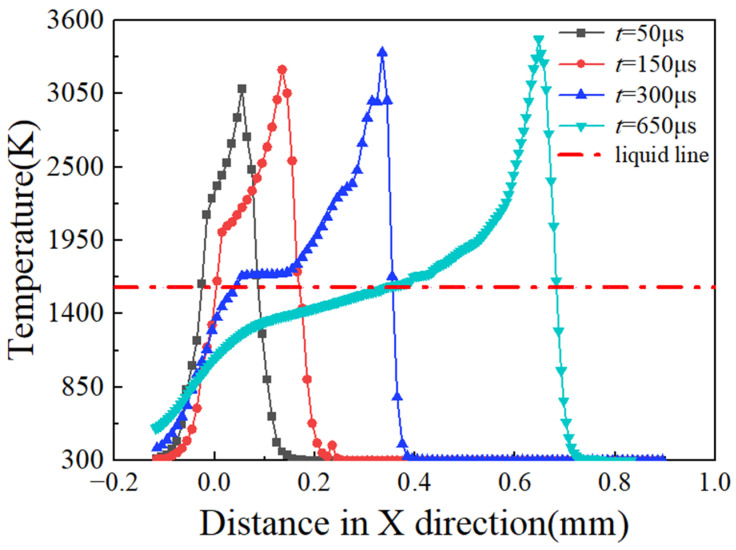
Temperature distribution along the X-axis at different times.

Figure 13 denotes the temperature distribution profile of the powder bed along the X-axis at different moments. The temperature field curve shows a steeper slope at the front end, indicating a higher temperature gradient. Similarly, by comparing the width of the isotherms in Figure 12, it can be observed that the front end is narrow, indicating a higher temperature gradient. This phenomenon occurs because as the heat source moves to the right, the powder layer is continuously preheated, resulting in a smaller temperature gradient and a wider isotherm at the rear end. Additionally, the material reaches a higher peak temperature due to advanced preheating through the conduction effect in the powder bed.

## 4. Relationship between Process Parameters and Molten Pool Morphology

### 4.1. The Effect of Scanning Speed

**Figure 14:**
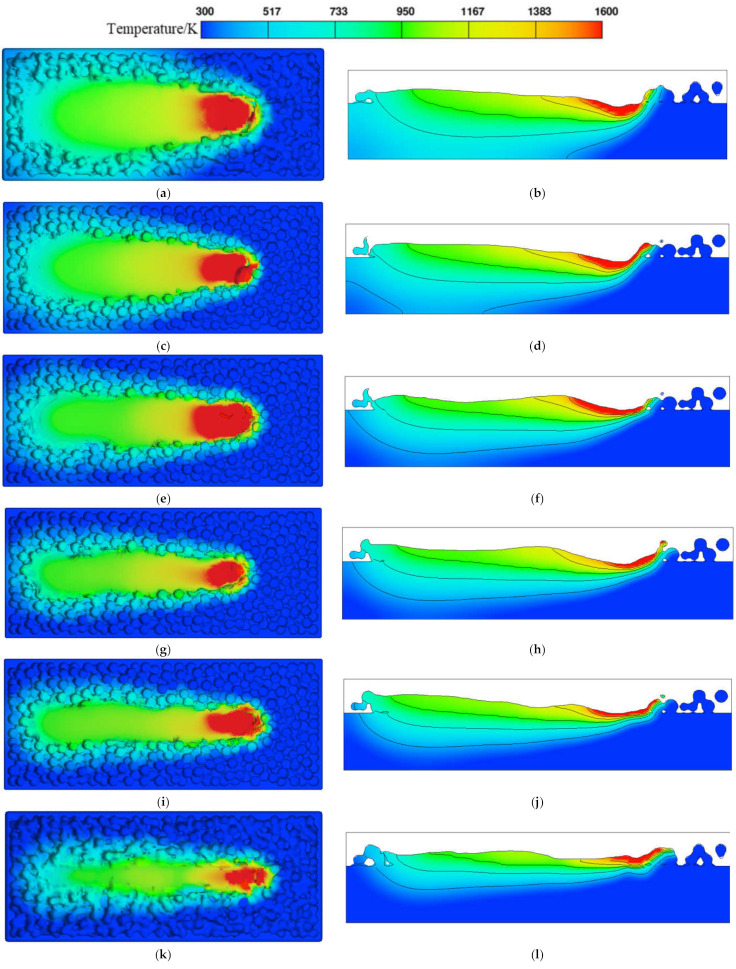
Temperature field of powder bed under different scanning speeds. (**a**) Three-dimensional temperature field at *v* = 500 mm/s, (**b**) temperature field in XZ section at *v* = 500 mm/s, (**c**) three-dimensional temperature field at *v* = 750 mm/s, (**d**) temperature field in XZ section at *v* = 750 mm/s, (**e**) three-dimensional temperature field at *v* = 1000 mm/s, (**f**) temperature field in XZ section at *v* = 1000 mm/s, (**g**) three-dimensional temperature field at *v* = 1250 mm/s, (**h**) temperature field in XZ section at *v* = 1250 mm/s, (**i**) three-dimensional temperature field at *v* = 1500 mm/s, (**j**) temperature field in XZ section at *v* = 1500 mm/s, (**k**) three-dimensional temperature field at *v* = 2000 mm/s, (**l**) temperature field in XZ section at *v* = 2000 mm/s.

**Figure 15:**
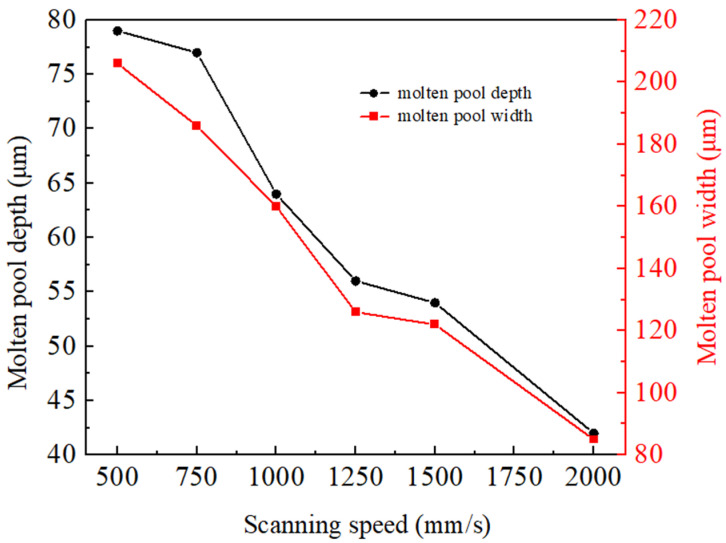
Molten pool size at different scanning speeds.

The effects of different scanning speeds (500, 750, 1000, 1250, 1500, and 2000 mm/s) on the temperature field and flow field were investigated at a laser power of 380 W, a powder layer thickness of 60 μm, and a laser spot diameter of 100 μm. Figure 14 shows the temperature field distribution of the powder bed and XZ section at X = 650 μm from the scanning start point. As the scanning speed increases, the width of the molten track tail decreases and the overall molten track width is evenly distributed. When the scanning speed is 500 mm/s, the width of the molten track is larger for two reasons. One is due to the scanning speed being small, the laser and powder action time being longer, and the surrounding material also being affected by melting; the second is due to the Marangoni effect in the molten metal like the scanned area behind the flow. When the scanning speed is 2000 mm/s, the width of the molten track is 80~90 μm, which is smaller than the laser spot diameter, and the molten track appears discontinuous. The width and depth dimensions of the molten pool at different scanning speeds are shown in Figure 15, where the dimensions of the molten pool decrease with the increase in scanning speed. The laser scanning speed is low, which causes the laser to stay on the powder for a longer time. As a result, the material absorbs more energy and a large area of the material reaches melting temperature, leading to a larger molten pool.

**Figure 16:**
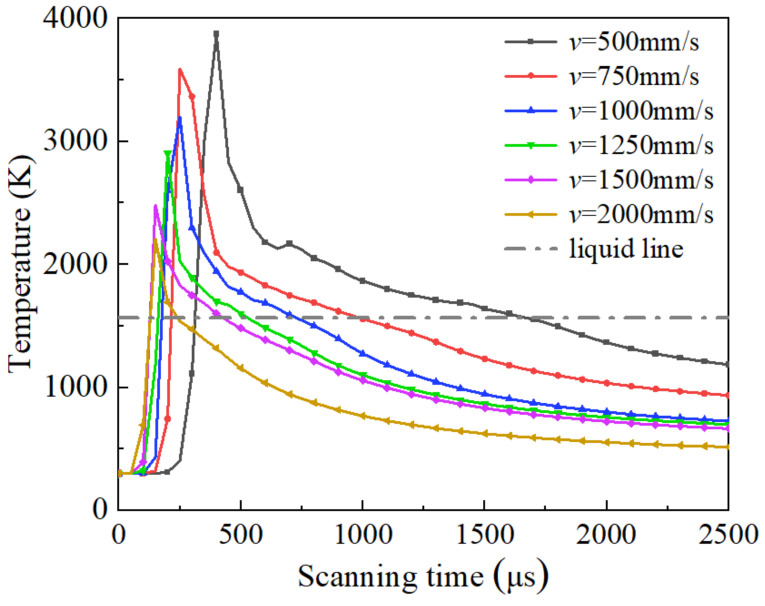
Evolution of characteristic node at different scanning speeds.

**Figure 17:**
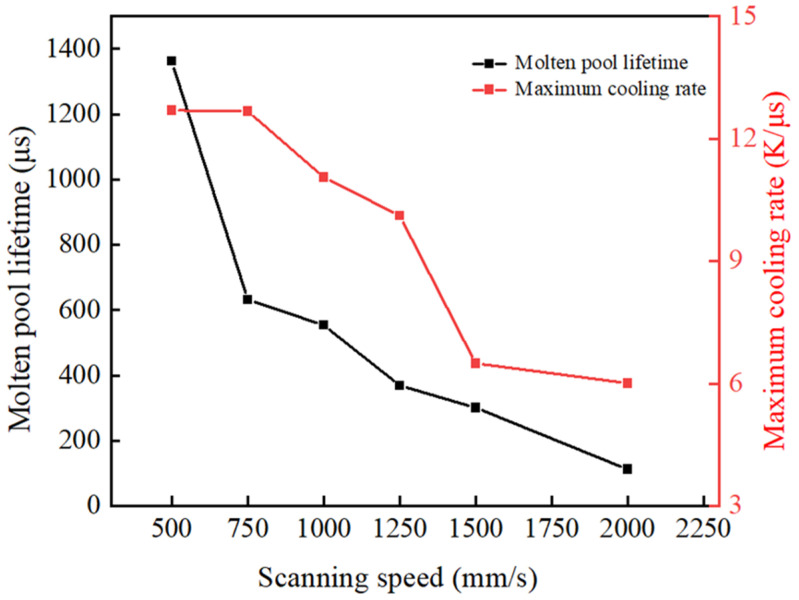
The maximum cooling rate and molten pool lifetime of the characteristic node under different scanning speed.

Figure 16 represents the relationship between the temperature and scanning time of the characteristic node (X = 200 μm, Y = 0 μm, Z = −20 μm) at different scanning speeds. The absolute value of the slope of the curve during temperature descent indicates the molten pool’s cooling rate. As shown in Figure 16, the faster the scanning speed, the shorter the time to reach the temperature peak at the characteristic node location, and the lower the temperature peak. This is because decreasing the scanning speed increases the powder’s absorption of energy, making it take more time to form a molten pool through the thermal diffusion of the energy [51]. Figure 17 shows the maximum cooling rate and molten pool lifetime of the characteristic node at different scanning speeds. The molten pool lifetime of a characteristic node is the duration for which the molten pool exists. This corresponds to the curve above the liquidus line in Figure 16. Decreasing the scanning speed prolongs the molten pool lifetime, leading to a fuller flow, as shown in Figure 17 [52]. Additionally, as the scanning speed decreases, the cooling rate increases due to the high-temperature material exchanging heat with the surrounding material.

**Figure 18:**
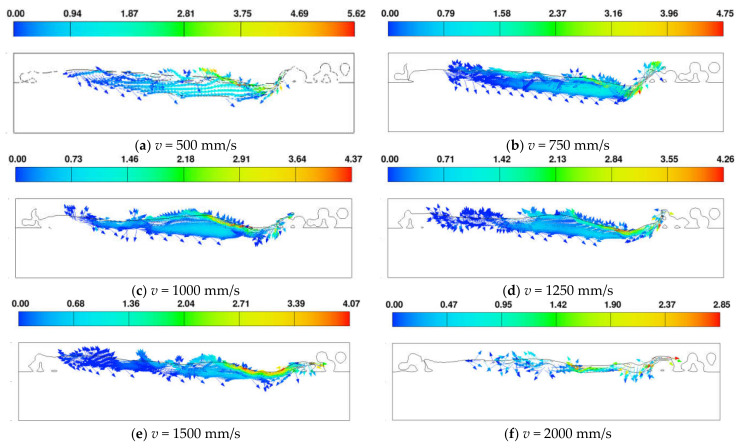
Velocity field of liquid metal flow at different scanning speeds.

Figure 18 illustrates the flow field in a ZY cross-section at a distance of X = 650 μm from the scanning starting point for various scanning speeds. The graph reveals a faster metal flow velocity at the rear of the molten pool and, as the scanning speed decreases, there is an increase in the maximum flow velocity of the molten pool. Concurrently, with an increase in scanning speed there is a reduction in the depth of the metal flow. Specifically, at a scanning speed of *v* = 2000 mm/s, there is intense flow observed only at the surface of the metal. This further emphasizes that with the increase in scanning speed, the molten pool’s depth decreases, and rapid solidification occurs within an extremely short timeframe.

### 4.2. The Effect of Laser Power

**Figure 19:**
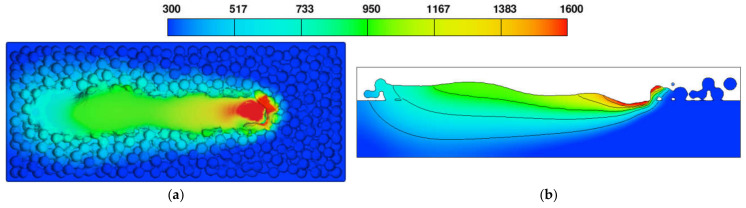

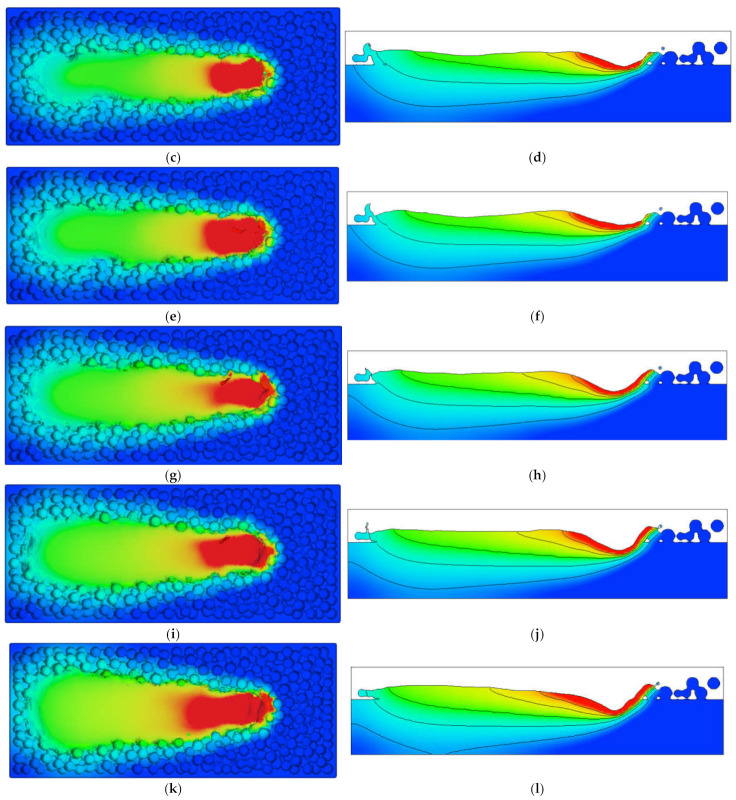
Temperature field of powder bed under different laser powers. (**a**) Three-dimensional temperature field at *P* = 280 W, (**b**) temperature field in XZ section at *P* = 280 W, (**c**) three-dimensional temperature field at *P =* 330 W (**d**) temperature field in XZ section at *P =* 330 W, (**e**) three-dimensional temperature field at *P =* 380 W, (**f**) temperature field in XZ section at *P =* 380 W, (**g**) three-dimensional temperature field at *P =* 430 W, (**h**) temperature field in XZ section at *P =* 430 W, (**i**) three-dimensional temperature field at *P =* 480 W, (**j**) temperature field in XZ section at *P =* 480 W, (**k**) three-dimensional temperature field at *P =* 580 W, (**l**) temperature field in XZ section at *P =* 580 W.

**Figure 20:**
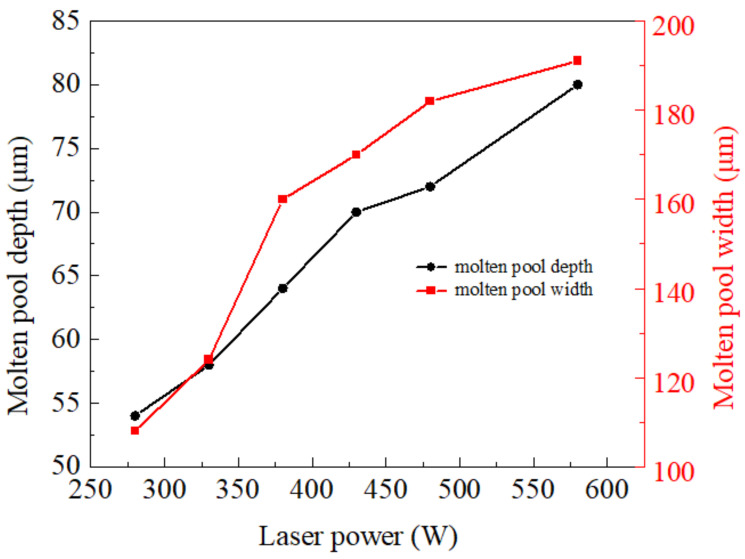
Molten pool size under different laser powers.

This study investigates the impact of different laser powers (280, 330, 380, 430, 480, and 580 W) on the temperature and flow fields seen at a scanning speed of 1000 mm/s, a powder layer thickness of 60 μm, and a laser spot diameter of 100 μm. It was found that the law of influence was opposite to that of the scanning speed. When *P* = 580 W, the power is higher and the molten pool track width is unevenly distributed, demonstrating a “pear-shaped” phenomenon. When *P* = 280 W, the power is lower and a discontinuous molten track tends to occur, as shown in Figure 19. In addition, with the increase in laser power, the size of the molten pool increases; the width and depth dimensions of the molten pools under different laser powers are shown in Figure 20.

**Figure 21:**
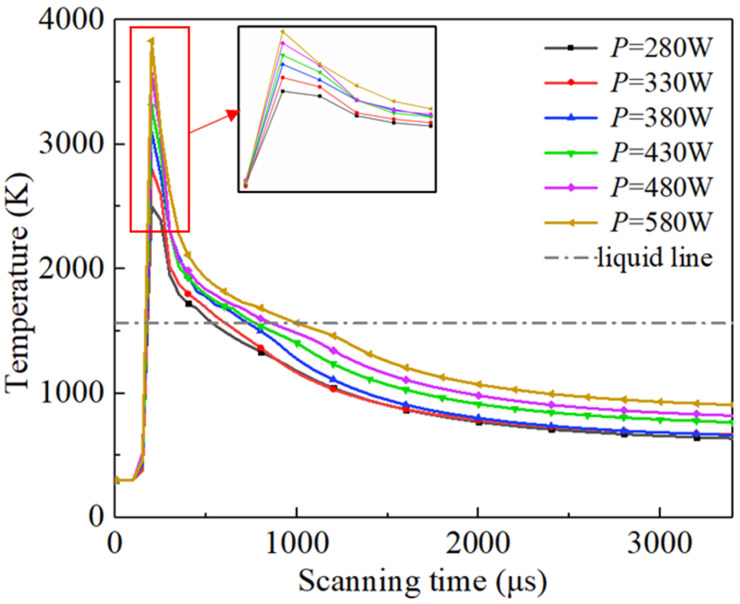
Evolution of characteristic node at different powers.

**Figure 22:**
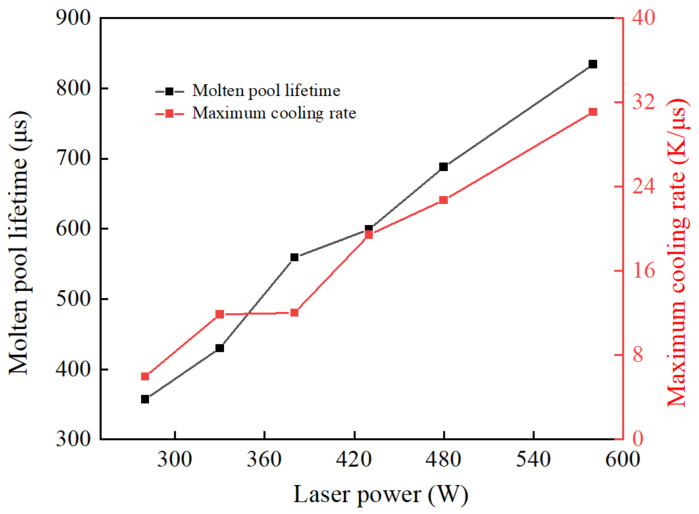
The maximum cooling rate and molten pool lifetime of the characteristic node under different laser power.

Figure 21 represents the relationship between the temperature and scanning time of the characteristic node (X = 200 μm, Y = 0 μm, Z = −20 μm) under different laser powers. Figure 22 shows the maximum cooling rate and molten pool lifetime of the characteristic node under different laser powers. Contrary to the law for obtaining scanning speed, an increasing laser power results in a longer molten pool lifetime and faster cooling rate. The fundamental reason for this is that a decrease in scanning speed and an increase in laser power both result in a significant increase in the powder bed temperature and a faster cooling rate, which have a similar effect on the morphology and size of the molten pool.

**Figure 23:**
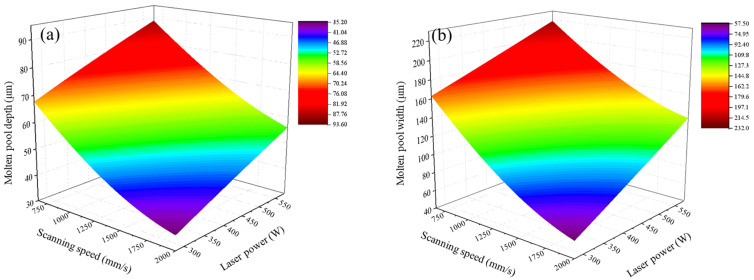
Response surface of the interaction effect of the laser power and scanning speed on (**a**) the depth of the molten pool and (**b**) the width of the molten pool.

In summary, with the decrease in scanning speed and increase in laser power, the molten pool size increases, as depicted by the response surface demonstrating their interactive effects in Figure 23. Furthermore, the molten track shape transitions from being discontinuous to gradually becoming uniform, ultimately exhibiting a “pear-shaped” morphology.

### 4.3. Window of Processing Parameters

**Figure 24:**
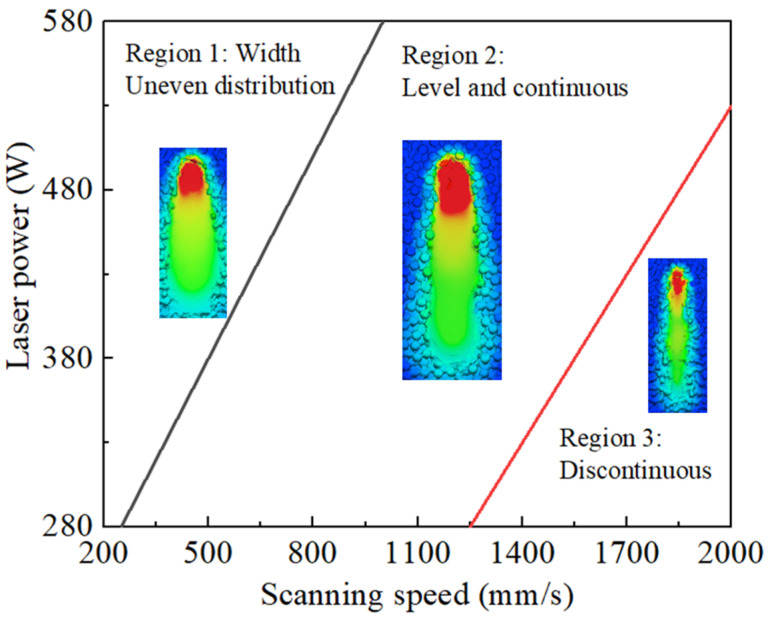
Parameter window for GH3625 based on its molten pool morphology.

Evaluation intervals were established from the effects of different laser powers and scanning speeds on the molten pool’s size and track. Under the process conditions of a high speed (*v* > 1500 mm/s) and low power (*P* < 330 W), issues such as an excessively narrow molten track, discontinuous molten track, and insufficient molten depth are prone to occur. Under the process conditions of a low speed (*v* < 750 mm/s) and high power (*P* > 330 W), the molten track has the phenomenon of a “pear shape” with uneven width distribution. Furthermore, low scanning speeds and high power parameters contribute to elevated temperatures in the molten pool, leading to severe material vaporization and resulting in decreased efficiency, with energy wastage. In order to visually display the process parameter ranges that correspond to an ideal forming quality, as well as the ranges associated with poor forming quality, a parameter window was established, as shown in Figure 24. Selecting process parameters based on this window allows us to achieve an optimal forming quality and provides suitable parameter ranges for subsequent research. For example, based on these preferable parameter ranges, the optimal process parameters for macro-scale formed parts can be studied. Process parameters with a higher power or slower scanning speeds result in higher stress values in the molten pool during solidification, leading to extensive crack formation [53]. Additionally, temperature field and stress field simulations can be conducted based on these preferable parameter ranges, and a quality prediction system for printed parts can be established [54]. Furthermore, directly applying these parameter ranges to practical production can reduce trial-and-error costs.

**Figure 25:**
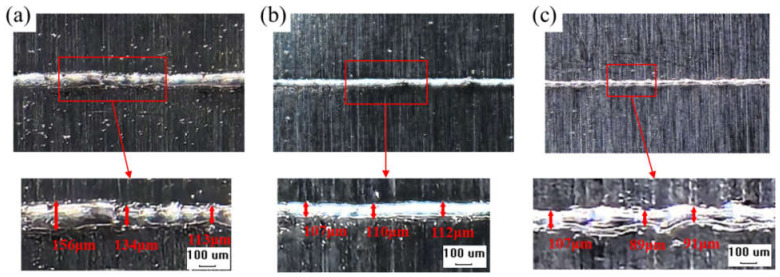
Single-track SLM experiments on GH3625 alloy: (**a**) *P* = 380 W, *v* = 500 mm/s; (**b**) *P* = 380 W, *v* = 1000 mm/s; (**c**) *P* = 280 W, *v* = 2000 mm/s.

Single-track SLM experiments on the GH3625 alloy were conducted, using the process parameters selected, on three designated regions of the parameter window. Figure 25 illustrates the results of these single-track SLM experiments. The molten track width was measured using Image-pro plus software 6.0. For region 1, a laser power of 380 W and a scanning speed of 500 mm/s were chosen, as depicted in Figure 25a. For region 2, a laser power of 380 W and a scanning speed of 1000 mm/s were selected, also shown in Figure 25a. Additionally, for region 3, a laser power of 280 W and a scanning speed of 2000 mm/s were utilized, as illustrated in Figure 25c. In region 1, the width distribution of the molten tracks is uneven, and an overheating phenomenon occurs on the surface due to an elevated molten pool temperature. In region 2, the molten track width is uniform and has a better surface morphology. In region 3, the molten track width is narrower and shows bending, with a tendency towards discontinuity. These findings are highly consistent with our simulation results, further demonstrating the accuracy of the model.

## 5. Conclusions

In this paper, a mesoscale model of the SLM process for the GH3625 alloy was established to simulate the forming process of a single molten track. The evolution process of the molten pool was revealed, and the effects of laser power and scanning speed on the molten pool size and molten track morphology were analyzed. A parameter window depicting the relationship between the molten pool’s morphology and process parameters was established using the simulation results. The conclusions can be summarized as follows:

(1) The material flows and deforms successively under primary driving forces such as steam recoil, Marangoni convection, surface tension, and gravity, ultimately forming a molten pool with a hill shape. Furthermore, as the laser heat source moves, the molten pool shape transitions from circular to elliptical, ultimately presenting a comet shape. The formation of this shape is due to the presence of surface tension gradients, causing the liquid-phase metal to reflux towards the already scanned region. The molten pool size of the simulation results matches the experimental results, with a 7% error.

(2) As the scanning speed decreases and the laser power increases, the molten pool depth and width increase due to the elevated central temperature and a larger temperature gradient. Moreover, the duration of the presence of the liquid-phase metal extends, accompanied by an increased flow velocity.

(3) At high scanning speeds (*v* > 1500 mm/s) and low power (*P* < 330 W) parameters, narrow and discontinuous molten tracks are formed, with a smaller molten pool depth and width. At low scanning speeds (*v* < 750 mm/s) and high power (*P* > 330 W) parameters, uneven width molten tracks are formed, with a larger molten pool depth and width.

## Figures and Tables

**Table 1:** Chemical composition of GH3625 (Wt%).

Elements	C	Cr	Mo	Fe	Nb	Al	Ti	Mn	Co	Si	N	Ni
Content	0.018	20.35	8.86	1.45	3.57	0.22	0.091	0.044	0.21	0.02	0.031	Bal

**Table 2:** Processing parameters and material properties.

Parameters	Value
Solidus temperature *T_m_*/K	1563
Liquids temperature *T_L_*/K	1623
Boiling temperature *T_v_*/K	3000
Surface tension at melting temperature *γ*_m_/(mN/m)	1906
Temperature coefficient of surface tension *dγ*/*Dt*/(mN∙m^−1^∙K ^−1^)	−0.37
Effective enthalpy of metal vapor Δ*H_v_*/(erg/g)	1.97 × 10^10^
Convective heat transfer coefficient *h*_con_/(W∙m^−2^∙K ^−1^)	80 [31]
Emissivity *ε*	0.8
Stefan–Boltzmann constant *σ_s_*/(W∙m^−2^·K^−4^)	5.67 × 10^−8^
Laser power *P*/W	280~580
Scanning speed *v*/(mm∙s^−1^)	500~2000
Laser spot diameter *d*/μm	100
Laser absorptivity *η*	0.38
Ambient temperature *T_ref_*/K	293.15
Shield gas	Argon
Environment pressure *p*_0_/Pa	700

## Data Availability

Data are contained within the article.
